# Personalized Fluoroscopic Angles in Watchman™ Left Atrial Appendage Closure Landing Zone Assessment: A Three-Dimensional Printed Simulation Study

**DOI:** 10.7759/cureus.8783

**Published:** 2020-06-23

**Authors:** Vikram Shee, Liwei He, Shenrong Liu, Xingfu Huang, Yanyu Chen, Liangzhen Xie, Xiaojiang Deng, Jian Peng

**Affiliations:** 1 Cardiology, Nanfang Hospital, Southern Medical University, Guangzhou, CHN

**Keywords:** watchman device, left atrial appendage closure, 3d printing, simulation, computed tomography, fluoroscopy

## Abstract

Background

Atrial fibrillation causes ischemic stroke when thrombi dislodge from a cardiac outpouching, the left atrial appendage (LAA), and embolize to the brain. LAA occlusion with the Watchman™ device (Boston Scientific Corporation, MA, USA), which prevents stroke, requires accurate LAA measurements for device sizing. We explore whether standard fluoroscopic LAA measurements improve when obtained at CT-derived viewing angles personalized to LAA anatomy while concurrently referring to three-dimensional (3D) CT.

Methods

Left atrial 3D reconstructions created from contrast CT (n=28) were analysed to identify personalized viewing angles wherein LAA dimensions (LAA maximum landing zone diameter and LAA length) were best observed. The 3D-CT reconstructions were then 3D printed with stands. Fluoroscopy of anatomically oriented models in the catheter lab simulated LAA angiography. Fluoroscopic images were acquired at standard (caudal 20˚/right anterior oblique 30˚) and personalized viewing angles. Repeated measurements of LAA dimensions were taken from CT (Control), fluoroscopy at standard angles (Standard), personalized angles (Blinded), and personalized angles while concurrently referring to 3D CT (Referred).

Results

Control measurements correlated and agreed better with Referred and Blinded measurements than with Standard measurements (diameter correlation and agreement: Control/Standard r=.554, limits of agreement [LOAs]=6.83/-5.91; Control/Blinded r=.641, LOA =5.67/-5.54; Control/Referred r=.741, LOA=4.69/-4.14; length correlation and agreement: Control/Standard r_s_=.829, LOA=9.61/-3.02; Control/Blinded r_s_=0.789, LOA=7.13/-4.94; Control/Referred r_s_=.907, LOA=4.84/-4.13). Personalized angles resulted in hypothetical device size predictions more consistent with Control (device size correlation: Control/Standard r_s_=.698, Control/Blinded r_s_=.731, Control/Referred r_s_=.893, P<0.001). False ineligibility rates were Standard=6/28, Blinded=6/28, and Referred=2/28.

Conclusion

This simulation suggests that personalized fluoroscopic viewing angles with in-procedural reference to 3D CT may improve the accuracy of LAA maximum landing zone diameter and length measurements at the Watchman landing zone. This improvement may result in more consistent device size selection and procedural eligibility assessment. Further clinical research on these interventions is merited.

## Introduction

Atrial fibrillation (AF) substantially increases the risk of cardioembolic stroke [1]. In non-valvular AF patients, stroke is often caused by thromboembolism from the left atrial appendage (LAA). Occluding the LAA in such patients can prevent stroke [2].

The Watchman™ device (Boston Scientific Corporation, MA, USA) is a commonly used transcatheter LAA occlusion device. Eligibility and device size selection for Watchman implantation depend on LAA dimensions, specifically, LAA landing zone diameter (measured from 1-2 cm anterior to the left upper pulmonary vein limbus to the inferior deflection of the LAA) and LAA maximum length along the implantation axis. LAA dimensions measured from fluoroscopy, contrast-enhanced cardiac CT, intracardiac echocardiography (ICE) and transesophageal echocardiography (TEE) are often discordant resulting in inaccurate device sizing [3]. The PROTECT AF trial required 1.8 device implantations per patient thus emphasizing the sizing dilemma [4].

CT LAA dimensions are obtained preprocedurally when the heart rate, rhythm and hydration status vary from the inprocedural state, resulting in non-representative results. Continuous TEE provides accurate and inprocedural dimensions, but is invasive and requires general anesthesia (GA) with associated risks. ICE is accurate, inprocedural and avoids GA; however, it is expensive, invasive and has limited views. Although fluoroscopic LAA dimensions are obtained inprocedurally and non-invasively, measurements are inaccurate compared to CT, TEE and ICE [3].

Fluoroscopic measurements of the anatomically variable LAA are taken at a standardized viewing angle, namely caudal 20˚ and right anterior oblique 30˚ (CAUD20/RAO30). CT-derived personalized fluoroscopic views have been proposed to improve measurement accuracy, yet have not been evaluated thus far [5].

Even with personalized views, the landing site selected on preprocedural CT may not be reselected on inprocedural fluoroscopy, perhaps because complex anatomy is poorly appreciated in two dimensions. We propose that inprocedural reference to three-dimensional (3D) CT during fluoroscopic analysis at personalized viewing angles can ensure measurements are made at comparable positions.

We investigate whether personalized fluoroscopic views and inprocedural reference to 3D CT can rectify fluoroscopic measurement errors. A simulation of LAA angiography using 3D printed left atrial (LA) models was developed due to the limited sample of Watchman cases in China.

## Materials and methods

Study protocol

A repeated measures experimental design was utilized. CT measurements of LAA dimensions, which accurately mirror model measurements, comprised the Control. Subsequently, three sets of fluoroscopic measurements were obtained, namely, LAA dimensions at (1) standard angles (Standard), (2) personalized angles (Blinded) and (3) personalized angles with concomitant reference to CT (Referred). LAA dimensions from each measurement category were used to assess eligibility for Watchman implantation, and to select a device size for implantation.

The correlation and agreement of the fluoroscopic measurements with Control measurements were explored. The clinical outcomes of the measurement techniques were investigated based on eligibility for Watchman implantation and predicted device size. Furthermore, contributors to measurement error (i.e., discrepancy between fluoroscopic measurements and Control measurements) were investigated using linear regression.

Inclusion and exclusion criteria

Non-valvular AF patients with indications for Watchman implantation who underwent cardiac CT angiography between 1 March 2016 and 31 July 2016 were included, regardless of whether Watchman implantation was performed (n=28). Patients with evidence of LAA thrombus preprocedurally, CHA_2_DS_2_VASc score <2, valvular AF, indications other than AF for long-term warfarin or absolute contraindications to warfarin were excluded prior to cardiac CT angiography. The CHA_2_DS_2_VASc score was calculated as follows: Congestive heart failure (1 point), Hypertension (1 point), Age >75 years (2 points), Diabetes Mellitus (1 point), Stroke (2 points), Vascular disease (1 point), Age 65-74 years (1 point), Sex category, i.e., female gender (1 point).

Image acquisition, processing and 3D printing

**Figure 1:**
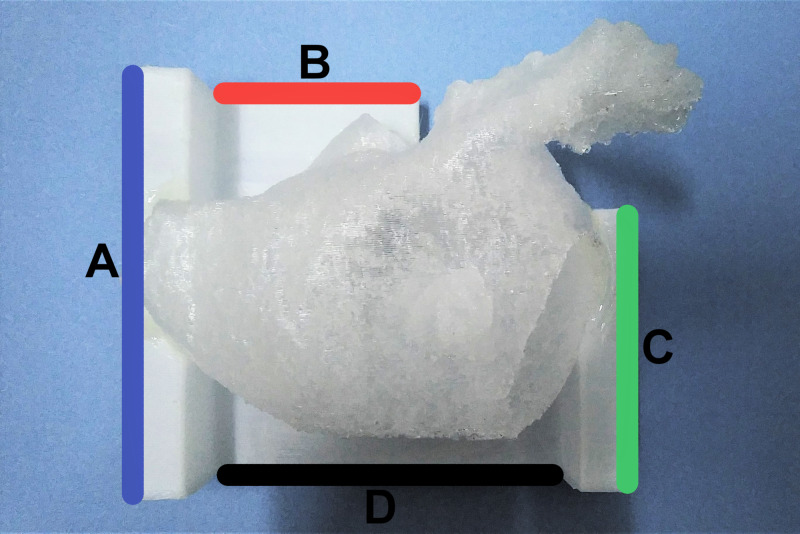
Orienting 3D printed models for simulated fluoroscopy The 3D printed models were oriented by aligning left (A, blue), superior (B, red), right (C, green) and inferior (D, black) edges of the stand to congruent edges of the operating table. An inferior reference line drawn on the table ensured consistent alignment between models. 3D, three dimensional.

CT DICOM® (Digital Imaging and Communications in Medicine) datasets (Medical Imaging & Technology Alliance, VA, USA) were acquired on a Philips Brilliance iCT 256-Slice CT scanner (78% R-R, 1-mm slice thickness; Philips Healthcare, OH, USA). 3D models of the LA and LAA were segmented using thresholding technique by a single experienced technician (3D Slicer 4.73) [6]. A customized stand oriented the LA identically to its CT position (Figure 1). Models were saved in a stereolithographic (STL) format and post-processed on Blender 3D (Blender Foundation, Netherlands) to thicken model walls inwards (preserving external dimensions), ensure manifold meshes and remove free vertices. STL files were converted to G-Code (Slic3r 1.2.9, lead developer: Alessandro Ranellucci, Italy) and printed on a fused deposition modeling 3D printer (Prusa i3; eMotion Tech, France) in high-impact polystyrene plastic (Public Color, China) at a 0.3-mm layer resolution. The printer was calibrated using digital calipers to be accurate to 100 µm ensuring print-outs were an exact representation of the digital 3D-CT surface model.

Simulation of fluoroscopy

**Figure 2:**
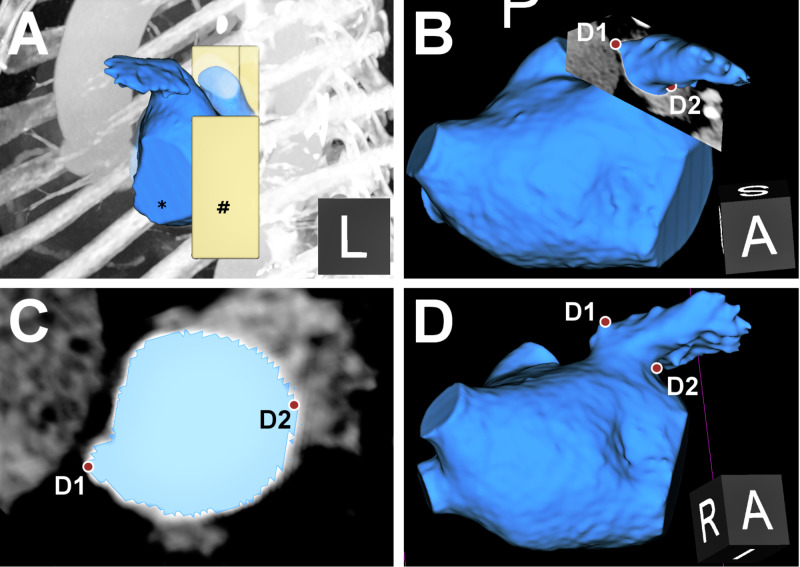
CT analysis and determination of personalized fluoroscopic angles (A) The left atrial appendage (*, blue) and personalized stand (#, yellow) are segmented from CT and reconstructed into a 3D model. The model is overlaid on a left lateral maximum intensity projection. A customized CT slice (multiplanar reconstruction) is cut across the target Watchman landing zone. Through inspection of 3D CT (B) and custom slice (C), the extent of the maximum LAA landing zone diameter is measured and marked with two fiducials (D1 and D2). (D) A 3D plane of view is selected wherein both fiducials are visible precisely at the borders of the LAA silhouette. Personalized fluoroscopic angles to replicate this view can be computed. When personalized angles are reproduced on fluoroscopy, an identical cardiac silhouette and landing zone to the one marked on CT should be observable. An analogous process was used to select personalized angles for maximum LAA length. 3D, three-dimensional; LAA, left atrial appendage.

**Figure 3:**
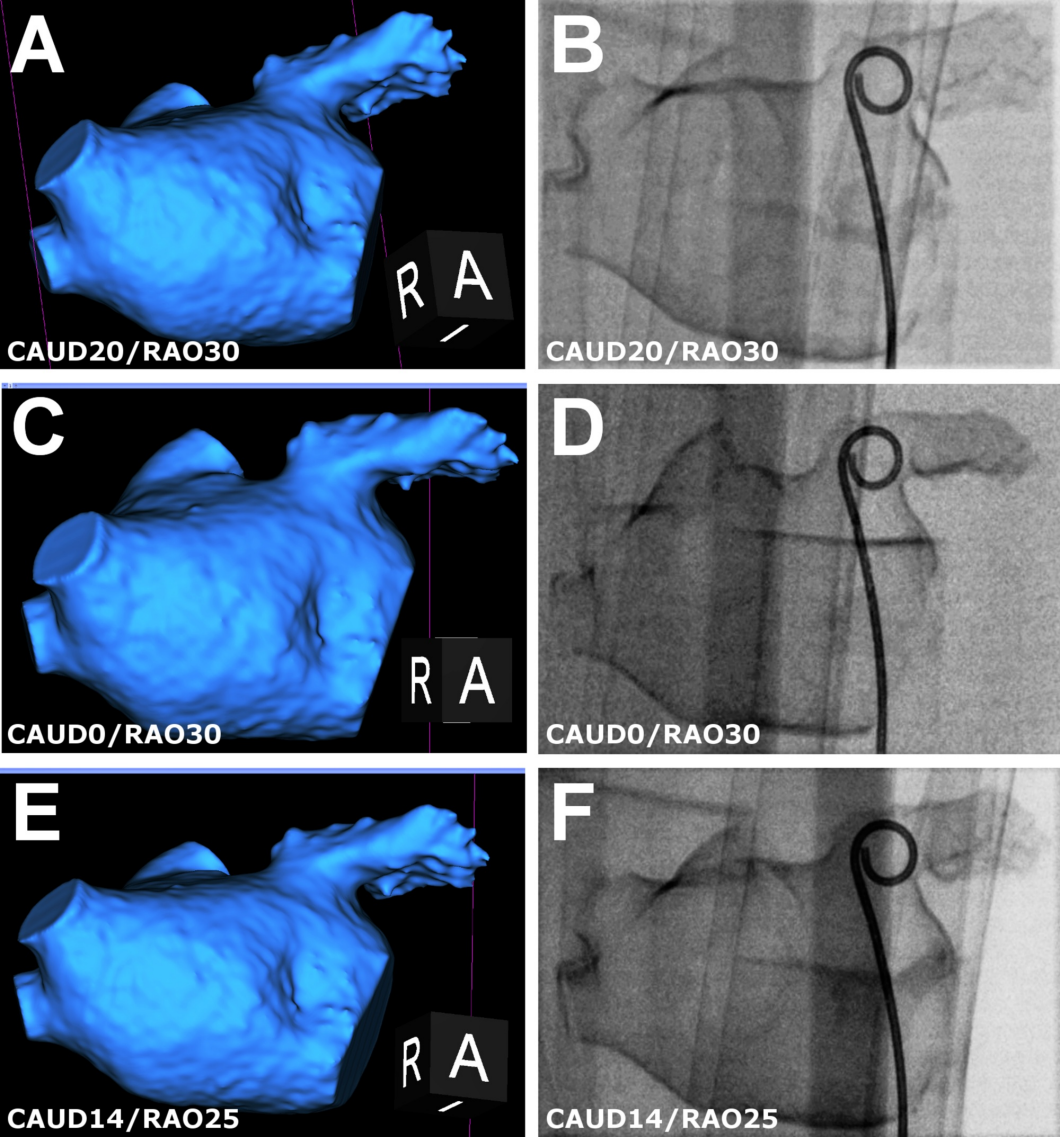
Personalized views in 3D CT and model fluoroscopy Optimal fluoroscopic views estimated on 3D CT (left) are successfully replicated on 3D printed LAA model fluoroscopy (right). The pigtail catheter in the LAA is for calibration (right). (A, B) Standard view (CAUD20/RAO30); (C, D) optimized for maximum length (CRAN0/RAO30); (E, F) optimized for maximum diameter (CAUD14/RAO25). 3D, three-dimensional; LAA, left atrial appendage; RAO, right anterior oblique; LAO, left anterior oblique; CAUD, caudal; CRAN, cranial.

Models were oriented anatomically on the surgical table in a standard catheter laboratory (AXIOM Sensis XP; Siemens, Germany) with a pigtail catheter within the LAA (6F, Infiniti Angiographic Catheter; Cordis, CA, USA). Subsequently, they were examined under the fluoroscope in a coronary mode at Standard (CAUD20/RAO30) and personalized angles (described below). Fluoroscopic measurements of LAA maximum diameter and length were made on the AXIOM workstation (Siemens) using the pigtail catheter for calibration (Figures 2, 3).

Image analysis

Step 1: Identification of Watchman Landing Zone

**Figure 4:**
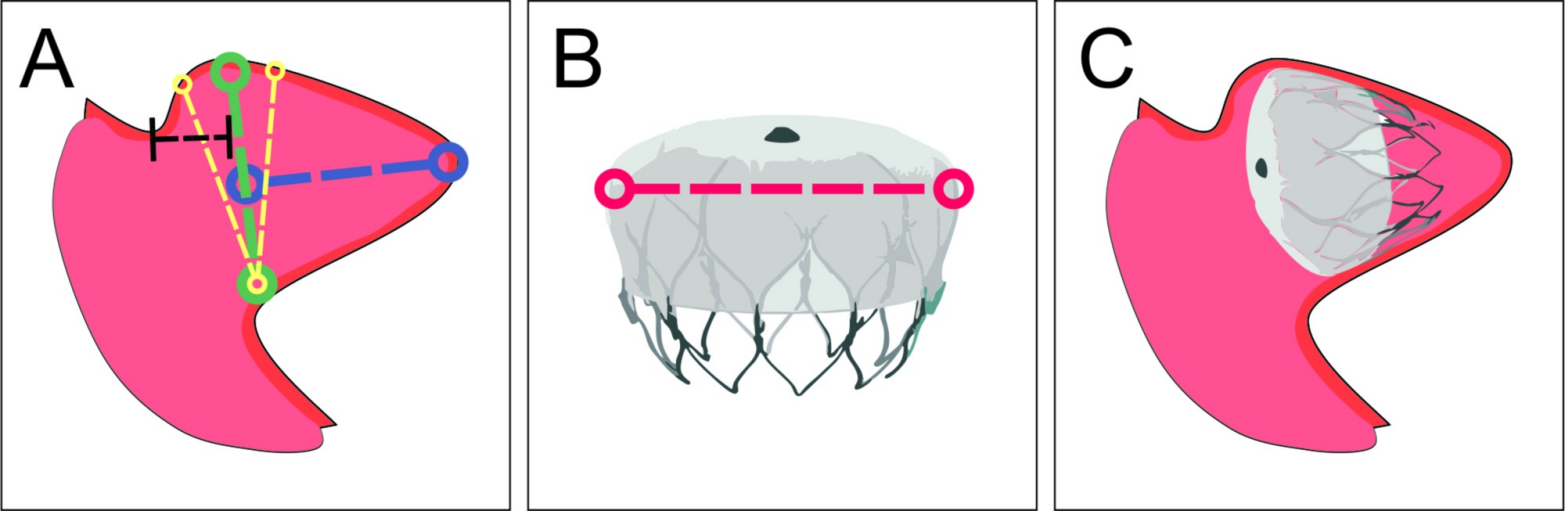
Device sizing (A) Measurement of LAA dimensions: LAA diameter is measured at the Watchman landing zone (green line), defined by a line from the inferior LAA deflection to a point 1-2 cm anterior to the LUPV limbus superiorly. Yellow lines mark the proximal and distal limits of the landing zone. The specific distance of the landing zone from the LUPV is selected to ensure perpendicularity with the LAA long axis. Herein, the optimal landing zone (green line) is selected at 1.5 cm from the LUPV (black line) because at this distance, the landing zone is perpendicular to the LAA long axis (blue line). Similarly, in other patient-specific LAA anatomies, the distance from the LUPV may be adjusted 1-2 cm to ensure the landing zone is oriented perpendicularly to the LAA long axis. The LAA length is measured from the landing zone center to the LAA tip, coaxial with the LAA cavity (blue line). The same definition is used for CT and fluoroscopic assessments. (B) Selecting a device size: Watchman device size represents the device diameter (red line). To secure the device, a size 8%-20% larger than the maximum LAA landing zone diameter is recommended. (C) Implantation: the figure shows orientation of an implanted Watchman device. LAA, left atrial appendage; LUPV, left upper pulmonary vein.

The area of the LAA at which the Watchman device would be expected to land was identified (Figure 4) [7].

Step 2: Derivation of Personalized Fluoroscopic Viewing Angles From CT

Figure 2 describes derivation of personalized fluoroscopic angles. At these angles, measurement points matching those on 3D CT can be measured on the fluoroscopic silhouette. Where feasible, a single fluoroscopic view for LAA maximum length and diameter was derived (n=8); otherwise two views optimized for either length or diameter measurements were derived (n=20) (Figure 3).

Step 3: CT and Fluoroscopic Measurements at Landing Zone

The maximum LAA diameter and length at the landing zone were measured with CT and fluoroscopy as illustrated in Figure 4A and detailed below:

1. Control measurements obtained from CT represented true model dimensions. During analysis, the segmentation label map and 3D model were overlaid on multiplanar reconstruction CT slices. This two-dimensional (2D)-3D hybrid method ensured measurements made from CT were accurate representations of the 3D surface model (Figure 2).

2. Standard measurements were obtained from model fluoroscopic images at a standard angle (CAUD20/RAO30).

3. Blinded measurements were obtained from model fluoroscopic images at personalized angles. During measurement, the operator was blinded from 3D CT.

4. Referred measurements were obtained on the reassessment of images at personalized fluoroscopic angles. Orthogonal 3D-CT views at personalized angles with the target landing zone marked were available for reference (Figures 2, 3).

Each fluoroscopic measurement was repeated three times with independent iterations of pigtail catheter calibration. Redundant measurements were averaged.

Other variables

Eligibility

Landing zone measurements were analysed to assess eligibility for Watchman implantation. Eligibility criteria included LAA maximum length ≥LAA landing zone diameter, and LAA landing zone diameter ≥17 mm and ≤31 mm (Figure 4) [8].

Device Size

If eligibility was established, a suitable device size (21, 24, 27, 30 or 33 mm) was selected with a target compression of 8%-20% at the LAA orifice (Figure 4). If two device sizes were feasible, the larger size was selected. Details are presented in manufacturer-supplied directions for use [8].

Contributors to Fluoroscopic Measurement Errors

**Figure 5:**
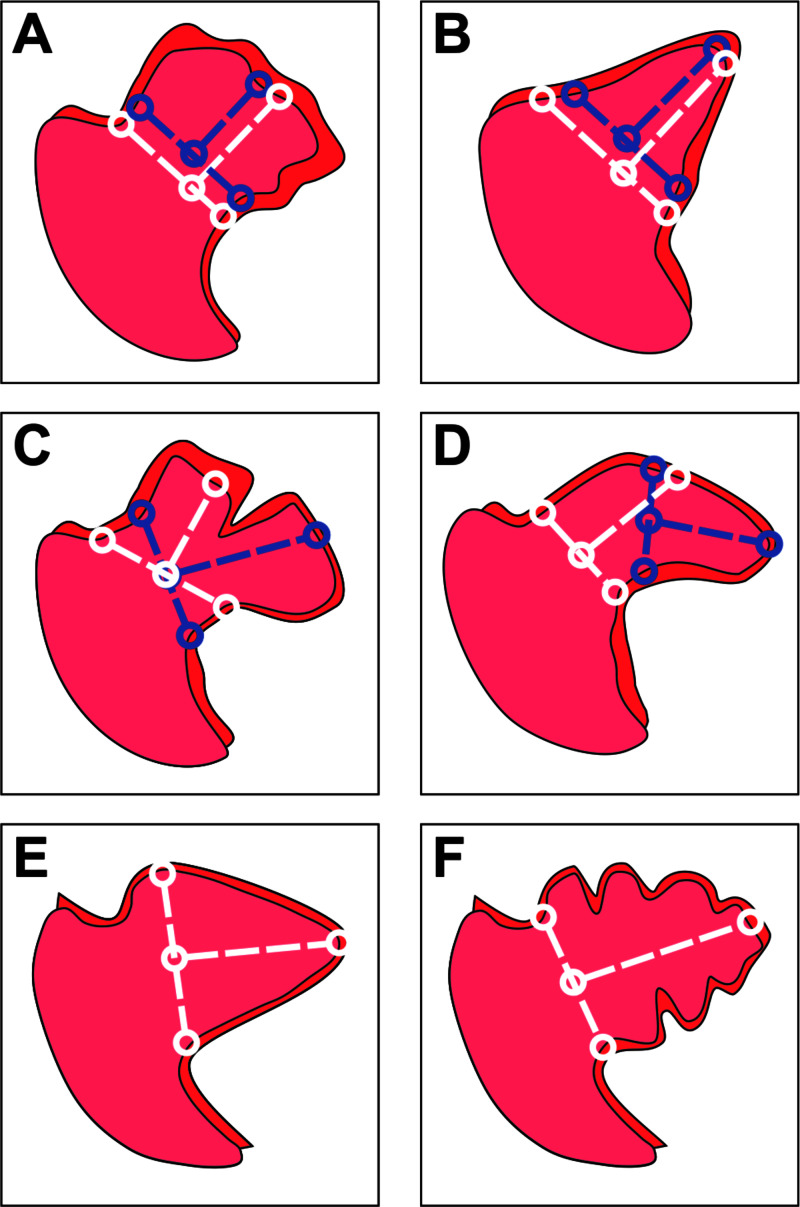
Left atrial appendage morphology and measurement error (A) Cauliflower morphology has unclear shoulders that make identifying landing zone landmarks difficult. (B) Cone morphology has steep gradients such that selecting a landing zone slightly proximally or distally would produce a much larger change in the measured landing zone diameter. (C) Bilobed morphology presents multiple feasible axes for implantation. (D) Chicken wing morphology presents multiple approaches for implantation, producing different measurements. (E) Windsock and (F) cactus morphologies present a single implantation axis; landing zone landmarks are distinct and thus measurement error is less likely.

The following contributors to the fluoroscopic measurement error were assessed: LAA morphology (windsock, cactus, cone, bilobed, cauliflower or chicken wing) was recorded, and selected morphologies that may cause inaccurate measurement (cone, bilobed, cauliflower and chicken Wing) were grouped for analysis (Figure 5) [9].

**Figure 6:**
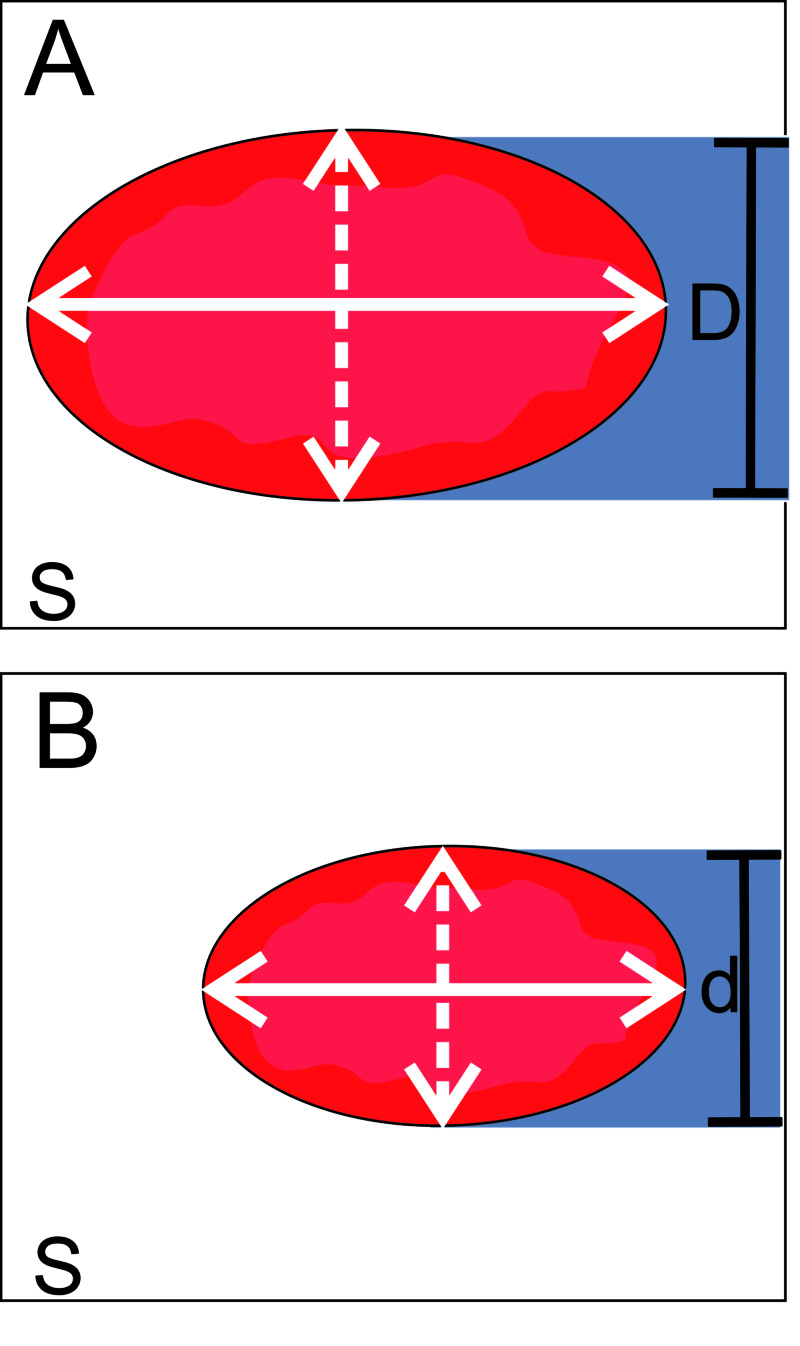
Contribution of eccentricity and absolute eccentricity to LAA measurement error The figure shows two oval LAA orifices of different sizes but identical shape casting fluoroscopic shadows (D, d) across their minimum landing zone diameter (dashed lines). When minimum diameter is measured instead of maximum diameter (solid lines) due to an inappropriate viewing angle, measurement error (minimum-maximum landing zone diameter) is greater in A than B. Eccentricities (1 - [short-axis LAA orifice diameter/long-axis diameter]) in A and B are identical, and fail to predict a greater measurement error in A. Absolute eccentricity (long-axis diameter - short-axis diameter) is greater in A than B, predicting greater measurement error in A. LAA, left atrial appendage.

Eccentricity (1 - [short-axis LAA orifice diameter/long-axis diameter]) and absolute eccentricity (long-axis LAA orifice diameter - short-axis diameter) were also recorded (Figure 6).

Bias reduction

De-identified image datasets were analysed in a randomized order and independent observers were assigned to each measurement category. Operators were blinded to whether a fluoroscopic image was optimized for diameter or length, so both were measured for each image.

Statistical analysis

Sample size (n=28) was selected to detect a medium effect size with a power of 0.8 at P<.05 for all statistics described unless stated otherwise (G-power; Heinrich Heine University Düsseldorf, Germany). Continuous variables were described as means ± standard deviations, and categorical variables as frequencies.

Pearson’s or Spearman’s coefficient assessed correlation between continuous variables. Bland-Altman (BA) plots assessed agreement variables. Fixed bias was assessed with one sample t-test and proportional bias with linear regression.

Repeated measures analysis of variance (rANOVA), Friedman’s ANOVA and Wilcoxon signed-rank tests were used as appropriate. Multiple linear regression (forward step-wise) analyses was performed to assess predictors of CT maximum diameter and length. Assumptions of regression were assessed as appropriate.

P<0.05 was considered significant. For multiple post hoc comparisons, Bonferroni correction was applied, and reference P values were stated. All statistical tests were performed on IBM SPSS Statistics, Version 24 (IBM Corp, Armonk, NY).

## Results

The average print time for a LAA model with the stand was 197 ± 48 minutes. The material cost (high-impact polystyrene) was 8.9 CNY (1.3 USD) per model. Patient baseline characteristics are defined in Table 1.

**Table 1:** Sample baseline characteristics ^1^Continuous variables are reported as means ± standard deviations, categorical variables are reported as frequency/total. ^2^HASBLED score was calculated as follows: Uncontrolled *H*ypertension (1 point), *A*bnormal liver (1 point) and/or renal function (1 point), *S*troke (1 point), *B*leeding tendency (1 point), *L*abile international normalization ratio (1 point), *E*lderly, i.e, >75 years (1 point), *D*rugs that predispose to bleeding or alcohol (1 point). ^3^CHA_2_DS_2_VASc score was calculated as follows: *C*ongestive heart failure (1 point), *H*ypertension (1 point), *A*ge >75 years (2 points), *D*iabetes mellitus (1 point), *S*troke (2 points), *V*ascular disease (1 point), *A*ge 65-74 years (1 point), *S*ex *c*ategory, i.e., female (1 point). RAO, right anterior oblique; LAO, left anterior oblique; CRAN, cranial; CAUD, caudal.

Variable	Frequency/total or mean ± standard deviation^1^	Percentage
Total number of cases	28	
Age	64.14 ± 10.52 yrs	
Sex		
Male	14/28	50%
Female	14/28	50%
Height	162.32 ± 8.10 cm	
Weight	63.55 ± 10.66 kg	
Body mass index	24.06 ± 3.13 kg	
Atrial fibrillation type		
Chronic	9/28	32.14%
Paroxysmal	19/28	67.86%
HASBLED score^2^	1.79 ± 0.738	
CHA_2_DS_2_VASc score^3^	3.18 ± 2.09	
Stroke/transient ischemic attack	5/28	17.86%
Coronary artery disease	6/28	21.43%
Congestive heart failure	9/28	32.14%
Diabetes mellitus	7/28	25.00%
Hypertension	13/28	46.43%
Impaired liver function	1/28	3.57%
Impaired kidney function	1/28	3.57%
Left ventricular ejection fraction	61.35 ± 7.46%	
Morphology		
Bilobed	3/28	10.71%
Cactus	4/28	14.29%
Cauliflower	1/28	3.57%
Chicken wing	3/28	10.71%
Cone	2/28	7.14%
Windsock	15/28	53.57%
Personalized angles		
RAO(+)/LAO(-)	27.85 ± 33.41	
CRAN(+)/CAUD(-)	14.35 ± 30.64	

**Figure 7:**
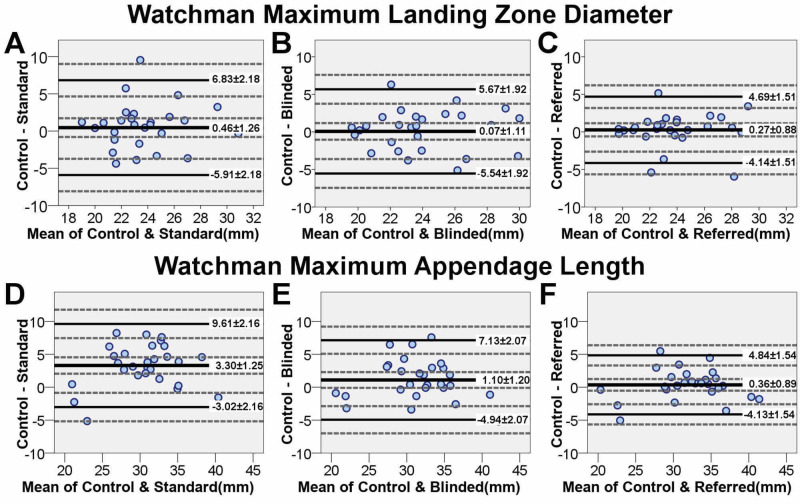
Bland-Altman plots of Control versus fluoroscopy measurements for maximum landing zone diameter and appendage length at the Watchman landing zone The figure represents the agreement of each set of fluoroscopic measurements with Control measurements as a baseline. The upper and lower limits of agreement for each fluoroscopic measurement represent the limits of underestimation and overestimation of Control measurements, respectively. Incremental narrowing of LOAs was apparent through (A) Standard, (B) Blinded and (C) Referred sets of fluoroscopic measurements. Upper LOA narrowed (reduced underestimation) through (D) Standard, (E) Blinded and (F) Referred measurements. Lower LOA marginally widened (increased overestimation) from Standard to Blinded, and Standard to Referred measurements. The mean discrepancy with Control decreased through Standard, Blinded and Referred measurements. LOA, limit of agreement. Mean discrepancy with Control is represented with a thick solid reference line; LOAs are represented with thin solid reference lines and 95% confidence intervals with dashed lines.

Control measurements of LAA maximum diameter and maximum length showed increasing correlation and narrowing limits of agreement with Standard, Blinded and Referred fluoroscopic measurements, respectively (maximum diameter: Control/Standard=.554 [P<.01], Control/Blinded r=.641 [P<.001], Control/Referred r=.751 [P<.001]; maximum length: Control/Standard r_s_=.829 [P<.001], Control/Blinded r_s_=.789 [P<.001], Control/Referred r_s_ =.907 [P<.001]; all one-tailed). BA agreement plots are illustrated in Figure 7. No proportional bias was detected. A significant fixed bias of 3.3 mm was detected for maximum length at Standard fluoroscopic angles.

Maximum diameter measurements did not differ significantly between the four measurement strategies (rANOVA, F(3, 81)=.345, P=.116). However, maximum length measurements were significantly different between measurement strategies (Friedman’s ANOVA, χ^2^(3) = 20.04, P<.001). Post hoc Wilcoxon’s signed rank test (significant at P<.0083 after Bonferroni correction) showed that Standard length measurements were significantly less than other measurement categories (P<.0083), while Control/Blinded (P=.047) and Referred/Blinded (P=.147) were similar.

Results of linear regression to predict the Control maximum diameter and maximum length are presented in Tables 2, 3. For Control maximum diameter, all assumptions of regression were met; however, for maximum length, mild heteroscedasticity was apparent.

**Table 2:** Regression summary: contribution of absolute eccentricity to error in the fluoroscopic measurement of maximum left atrial appendage diameter b represents unstandardized regression coefficients; SE represents standard error of b. *P<.05, **P<.01, ***P<.001. ^1^Outcome variable: Control maximum diameter measurements (ground truth). ^2^Absolute eccentricity = Control maximum left atrial appendage orifice diameter – Control minimum left atrial appendage orifice diameter.

	Standard^1^		Blinded^1^		Referred^1^	
	Step 1^**^	Step 2^***^	Step 1^***^	Step 2^***^	Step 1^***^	Step 2^***^
Predictor variables	b (SE)	b (SE)	b (SE)	b (SE)	b (SE)	b (SE)
Fluoroscopic maximum diameter	0.497^**^ (0.147)	0.444^**^ (0.131)	0.595^***^ (0.140)	0.505^**^ (0.136)	.0775^***^ (0.134)	0.697^***^ (0.121)
Absolute eccentricity^2^		0.428^**^ (0.148)		0.336^*^ (0.149)		0.346^**^ (0.119)
Coefficient of determination (R^2^)	.307	.481	.411	.511	.564	.674
Change in R^2^ (ΔR^2^)		0.174		.100		.110
No. of cases (n)	28					

**Table 3:** Regression summary: contribution of selected morphologies to error in the fluoroscopic measurement of maximum left atrial appendage length b represents unstandardized regression coefficients; SE represents standard error of b. *P<.01, **P<.001 ^1^Outcome variable: Control maximum length measurements (ground truth). ^2^Selected morphologies include cactus, cauliflower, chicken wing, cone and bilobed. ^3^Step 2 for Referred measurements was excluded since selected morphologies did not add any significant predictive value.

	Standard^1^		Blinded^1^		Referred^1,3^
	Step 1^**^	Step 2^**^	Step 1^**^	Step 2^**^	Step 1^**^
Predictor variables	b (SE)	b (SE)	b (SE)	b (SE)	b (SE)
Fluoroscopic maximum length	0.875^**^ (0.136)	0.708^**^ (0.143)	0.785^**^ (0.104)	0.655^**^ (0.103)	0.925^**^ (0.089)
Selected morphologies^2^		-3.295^**^ (1.377)		-3.294^*^ (1.168)	
Coefficient of determination (R^2^)	.616	.687	.689	.764	.805
Change in R^2^ (ΔR^2^)		.071		.075	
No. of cases (n)	28				

Watchman ineligibility rates were CT=5/28, Standard=7/28, Blinded=7/28 and Referred=3/28. False ineligibility rates were Standard=6/28, Blinded=6/28 and Referred=2/28. Among eligible patients, Control predicted device sizes significantly correlated with fluoroscopic sizes (device size: CT/Standard r=.695, CT/Blinded r=.731, CT/Referred r=.893, P<.001, one-tailed).

## Discussion

To the best of our knowledge, this simulation is the first to suggest improved accuracy of Watchman landing zone measurements through personalized fluoroscopic angles and in-procedural reference to 3D CT. Herein, we verify feasibility and hypothesize clinical benefit of these methods, justifying further clinical investigation. 

Fluoroscopy in Watchman implantation

Fluoroscopy allows real-time, non-invasive and in-procedural imaging. However, in Watchman implantation, LAA fluoroscopic measurements are inaccurate compared to CT, TEE or ICE limiting its dependability for device sizing. While cardiac CT provides useful measurements, being preprocedural, it does not capture temporal changes in hydration status, heart rate and rhythm that can affect in-procedural LAA dimensions. While continuous TEE is an accurate alternative, it is invasive and requires GA. ICE is at par with TEE regarding measurement accuracy and clinical outcomes, is in-procedural and avoids GA; however, it is invasive, expensive and has limited views. Therefore, improving fluoroscopic measurements is of great importance.

**Figure 8:**
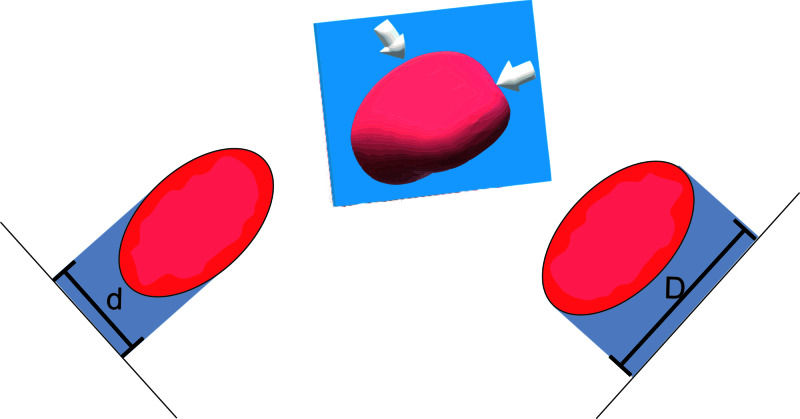
Mechanism by which Standard fluoroscopic angles may induce measurement error Fluoroscopic shadows (measuring D and d) of the same LAA at the Watchman landing zone (blue plane) when viewed at two different angles (white arrows) produce significantly different LAA landing zone diameter measurements (D>d). Incorrect fluoroscopic viewing angles can result in failure to observe the maximum LAA landing zone diameter. LAA, left atrial appendage.

Previous research demonstrated that CT-based personalized fluoroscopic angles benefit aortic valve measurements for TAVI [10]. Figure 8 illustrates how personalized angles can be beneficial. Herein, the same hypothesis is tested for LAA measurements.

It is difficult to gauge intricate anatomy from the 2D fluoroscopic silhouette. 3D-CT registration on fluoroscopy during Watchman LAAC has been demonstrated to tackle this constraint [11]. Similarly, we referred to 3D-CT images during fluoroscopy, but while utilizing personalized viewing angles concurrently.

The clinical impact of measurement improvements is investigated through hypothetical device size selection and procedural eligibility assessment. Mechanisms by which interventions improve fluoroscopic measurements are investigated through regression analysis.

Simulations of Watchman implantation

Otton et al. first described Watchman device size prediction by implantation into a flexible 3D printed LAA model derived from CT [12]. Subsequent clinical studies have established this method in device sizing [13]. A 3D printed phantom simulation of LAA fluoroscopic angiography, established previously by Sra et al., is utilized herein to test interventions to improve fluoroscopic measurement accuracy [14].

Measurement accuracy of personalized fluoroscopy

This simulation demonstrates personalized fluoroscopic viewing angles and reference to 3D-CT images during fluoroscopy (Referred measurement technique) improves fluoroscopic measurement accuracy (i.e. increased correlation and narrowed limits of agreement of fluoroscopic LAA measurements with Control). Incremental improvements support concomitant use of both interventions (Figure 7, Tables 2, 3).

Underestimating LAA landing zone diameter results in device undersizing leading to reduced stability and possible device embolization. The Referred measurement technique was less likely to underestimate the LAA landing zone diameter and may speculatively reduce device embolization (represented by a 2.14-mm narrowing in the upper LOA between Standard and Referred measurements of maximum diameter) (Figure 7). Furthermore, the increased measurement accuracy with Referred measurements resulted in more consistent device size selection with Control (device size correlation: Control/Referred rs=.893 vs. Control/Standard rs=.695, P<0.001).

If the LAA length cannot accommodate the length of the Watchman device, a patient is considered ineligible for implantation. Underestimation of maximum length was reduced with Referred measurements as compared to the Standard technique, represented by a 4.77-mm narrowing in the upper LOA between the two. This may prevent patients from incorrectly being excluded from Watchman LAAC due to inaccurate length measurements (false exclusions: Standard=6/28, Blinded=6/28, Referred=2/28) [8].

In our sample, improvements in device size prediction and reductions in false exclusions support replacement of the Standard measurement technique with the Referred technique. Improvements in length measurements, but not diameter measurements are generalizable based on 95% confidence intervals of LOA (Figure 7) [15].

Contributors to measurement error

${\rm Measurement}_{Control}={\rm Measurement}_{Fluoroscopy}+Measurement\ Error$

Through linear regression, contributors to fluoroscopic measurement error and how interventions mitigated error were investigated.

${LAA\ Diameter}_{Control}={LAA\ Diameter}_{Fluoroscopy}+{\rm Error}_{Eccentricity}+\ {\rm Error}_{unknown}$

The contribution of LAA landing zone eccentricity and absolute eccentricity to maximum diameter measurement error was explored. In our sample, eccentricity did not contribute to measurement error. Speculatively, while appendages differing greatly in size may share the same eccentricity, the maximal measurement error due to fluoroscopic views would be greater for a larger appendage (Figure 6).

${LAA\ Diameter}_{Control}={LAA\ Diameter}_{Fluoroscopy}+{\rm Error}_{Absolute\ Eccentricity}+\ {\rm Error}_{unknown}$

Absolute eccentricity significantly contributed to measurement error at Standard fluoroscopic angles (ΔR^2^=17.4%).

Personalized fluoroscopic angles optimized for the LAA maximum osital diameter reduced error due to absolute eccentricity (Blinded ΔR^2^=10%). Improved measurement accuracy observed with additional reference to 3D CT (Referred ΔR^2^=11%) was unrelated to absolute eccentricity and was possibly attributable to the consistent selection of measurement points between 3D CT and fluoroscopy (Table 2).

${LAA\ Length}_{Control}={LAA\ Length}_{Fluoroscopy}+{\rm Error}_{Morphology}+\ {\rm Error}_{unknown}$

The 2D visualization of LAA morphology complicates accurate fluoroscopic measurements by making selection of implantation location, direction and strategy difficult. Selected morphologies that are challenging to measurements are described in Figure 5. Selected morphologies contributed equally to measurement errors for Standard and Blinded measurements. The Referred measurement technique mitigated morphology-induced error, perhaps by improving anatomical appreciation of the 2D LAA silhouette (Table 3).

Limitations

This study is limited as it is a simulation, and consecutive sampling was used. Simulation allowed us to evaluate feasibility, provide proof of concept and hypothesize benefit despite a limited sample of Watchman implantations in China.

Differences between this simulation and a clinical setting merit consideration. CT measurements herein closely mirror 3D printed model measurements (through printer calibration), and are the Control for comparisons with fluoroscopic measurements.

Herein, cardiac CT was acquired at 78% R-R (for compatibility with the Carto XP system; Biosense Webster, Inc., CA, USA), while ventricular systole captures largest LAA dimensions. The simulated fluoroscopic silhouette is assumed to represent maximum LAA dimensions, and systematic error due to cardiac phase is rectified by the repeated measures design. Clinically, imaging a standardized cardiac phase between modalities is more important since a dynamic heart is imaged rather than a static model.

As this was a simulation, clinical variables are not available. Comparisons between CT, TEE and personalized fluoroscopic measurements and their effects on device sizes selected, percentage of successful implantations and number of devices deployed require further investigation.

## Conclusions

The results of this simulation study of LAA angiography in Watchman device implantation show that personalized fluoroscopic viewing angles and in-procedural reference to 3D CT may incrementally improve the accuracy of fluoroscopic measurements of LAA dimensions, as compared to standard fluoroscopic angles. Improved measurement accuracy will result in improved consistency in device size predictions and procedural eligibility assessment. Based on this evidence, further clinical evaluation of these methods is indicated.
